# Opening Wedge Osteotomy for Valgus Deformity of the Little Finger after Proximal Phalangeal Fracture in Children: Two Case Reports

**DOI:** 10.1155/2018/1526054

**Published:** 2018-04-17

**Authors:** Souichi Ohta, Ryosuke Ikeguchi, Hiroki Oda, Hirofumi Yurie, Hisataka Takeuchi, Shuichi Matsuda

**Affiliations:** Department of Orthopaedic Surgery, Kyoto University, Kyoto, Japan

## Abstract

In the treatment of posttraumatic valgus deformity of the pediatric little finger, it is usually difficult to achieve accurate correction of angular and rotational deformity using closing wedge osteotomy. We report two cases of valgus deformity of the little finger (both 11-year-old female patients) successfully treated using opening wedge osteotomy followed by intramedullary semirigid fixation with a single Kirschner wire. A wire tip inserted from the retrocondylar fossa of the proximal phalangeal head was advanced along the radial side of the intramedullary cortex after gradual opening of the osteotomy site. If needed, further fine adjustment of the rotational alignment can be performed even after K-wire insertion. Postoperatively, the gap between the little and ring fingers in the fully extended and adducted position and the finger overlapping in the fully flexed position were completely resolved. The flexibility of the pediatric bone and sagittal clearance between the wire and the inner wall of the proximal phalangeal medullary cavity allow fine adjustment of the rotational alignment even after wire insertion.

## 1. Introduction

A valgus deformity of the little finger sometimes occurs after conservative treatment or neglect of the proximal phalangeal neck fracture. Because of remaining ulnar tilt of the proximal phalangeal distal articular surface with or without rotation, the distal part of the little finger cannot touch the ulnar side of the ring finger in the fully extended and adducted finger position, although the proximal parts of the fingers can touch. The ring and little fingers overlap when the patient makes a fist, causing functional impairment [1].

To resolve the problems associated with such valgus deformity, closing wedge osteotomy is usually performed with fixation using a plate and screws or two Kirschner wires (K-wires) [2–4]. However, it is difficult to accomplish the exact resection of wedge bone and fixation according to preplanned correction angles. In contrast, an opening wedge osteotomy needs only one cutting line and careful opening of the wedge while making the opposite side cortex the center of rotation of angulation [5]. As pediatric bones have a more elastic cortex and thicker periosteum than adult bones, opening wedge osteotomy is suitable for the treatment of pediatric bone deformity; moreover, children have superior bone healing potential and do not necessarily need bone grafting into the opening gap at the osteotomy site. After opening the osteotomy site, we performed intramedullary fixation with a single K-wire. This semirigid fixation allows further fine adjustment of the rotational alignment even after K-wire insertion. Herein, we report two cases of pediatric little finger posttraumatic valgus deformity successfully treated with opening wedge osteotomy followed by intramedullary fixation with a single K-wire.

## 2. Case Reports

### 2.1. Surgical Procedure

**Figure 1:**
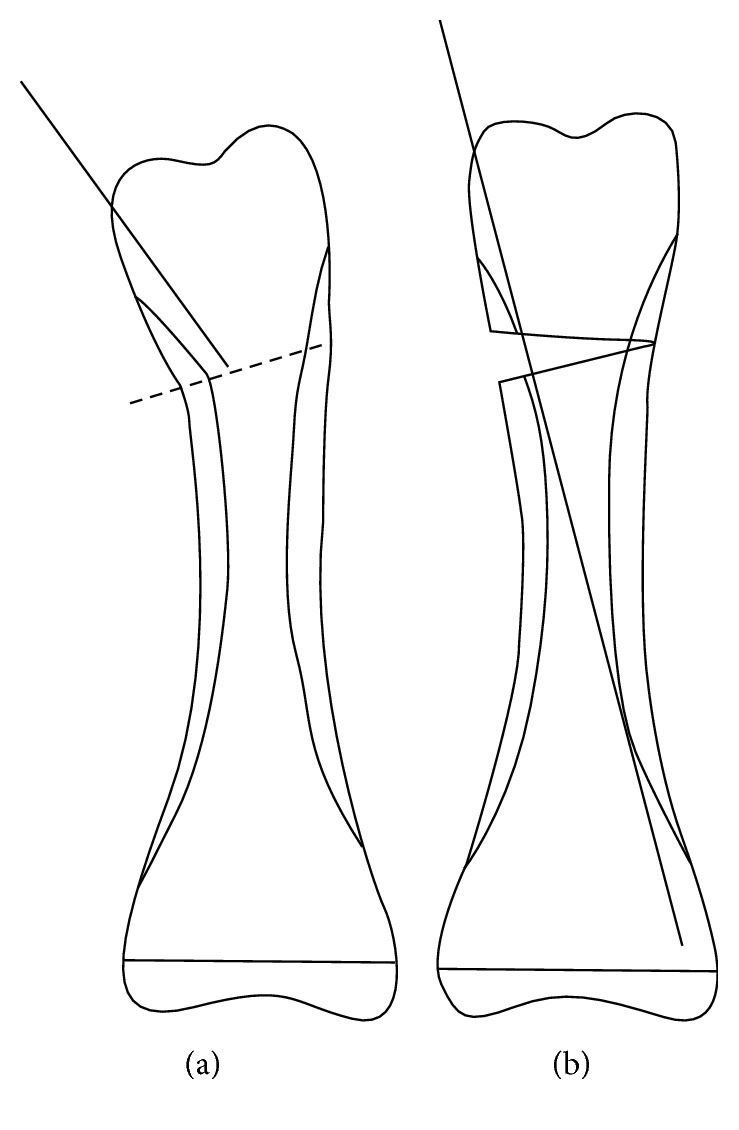
Surgical procedure. (a) Insert a K-wire until just distal to the preplanned osteotomy line (a dotted line). (b) Advance the tip of the K-wire along the radial side of the intramedullary cortex after gradual opening of the osteotomy site.

A midlateral skin incision of approximately 1 cm was made over the distal ulnar side of the proximal phalanx. The ulnar lateral band was moved aside dorsally, and the distal ulnar side of the proximal phalanx was exposed subperiosteally. An image intensifier was used to confirm the preplanned osteotomy line at the distal metaphysis of the proximal phalanx. The osteotomy line does not need to be the same as the fracture line, as long as the center lines of the reconstructed proximal phalanx and the middle phalanx of the same digit coincide. The osteotomy line in the metaphysis was preplanned to be parallel with a line tangential to the distal articular surfaces on an anteroposterior view on plain radiography. Multiple drilling was made at the preplanned osteotomy line using a 1 mm diameter wire. A 1.2 mm K-wire was then inserted through the retrocondylar fossa of the proximal phalangeal head, and the K-wire tip was advanced until just distal to the preplanned osteotomy line (Figure 1(a)). The insertion angle between the K-wire and the distal ulnar cortex of the phalangeal head was approximately 20°. Osteotomy was performed with a small thin osteotome, leaving the most radial cortex of the osteotomy site intact. The osteotomy site was then gradually opened until the preplanned angle was achieved, and the tip of the K-wire was advanced along the radial side of the intramedullary cortex to a point just distal to the epiphyseal line (Figure 1(b)). The distal tip of the exposed K-wire was bent, truncated, and buried under the skin. Bone grafting was not performed. After closing the surgical incision, the little finger was loosely buddy taped with the ring finger to allow active range of motion (ROM) exercises. At postoperative 4 weeks, we removed the K-wire after radiographic confirmation of callus formation at the osteotomy site.

### 2.2. Case 1: An 11-Year-Old Female

At 9 years of age, the patient fractured the proximal phalanx of the right little finger during kendo (Japanese fencing) training. At the time of the injury, she felt pain but did not consult any medical institution. When the pain resolved, the patient noticed valgus deformity of the little finger in extension and difficulty in gripping caused by finger overlapping.

**Figure 2:**
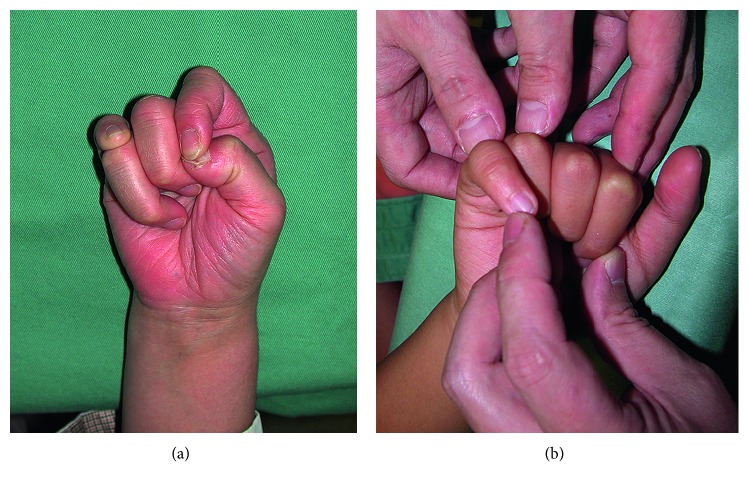
Preoperative photographs of the affected hand in Case 1. (a) Making a fist was difficult due to finger overlapping. (b) Passive finger flexion under general anesthesia revealed marked rotation of the little finger.

**Figure 3:**
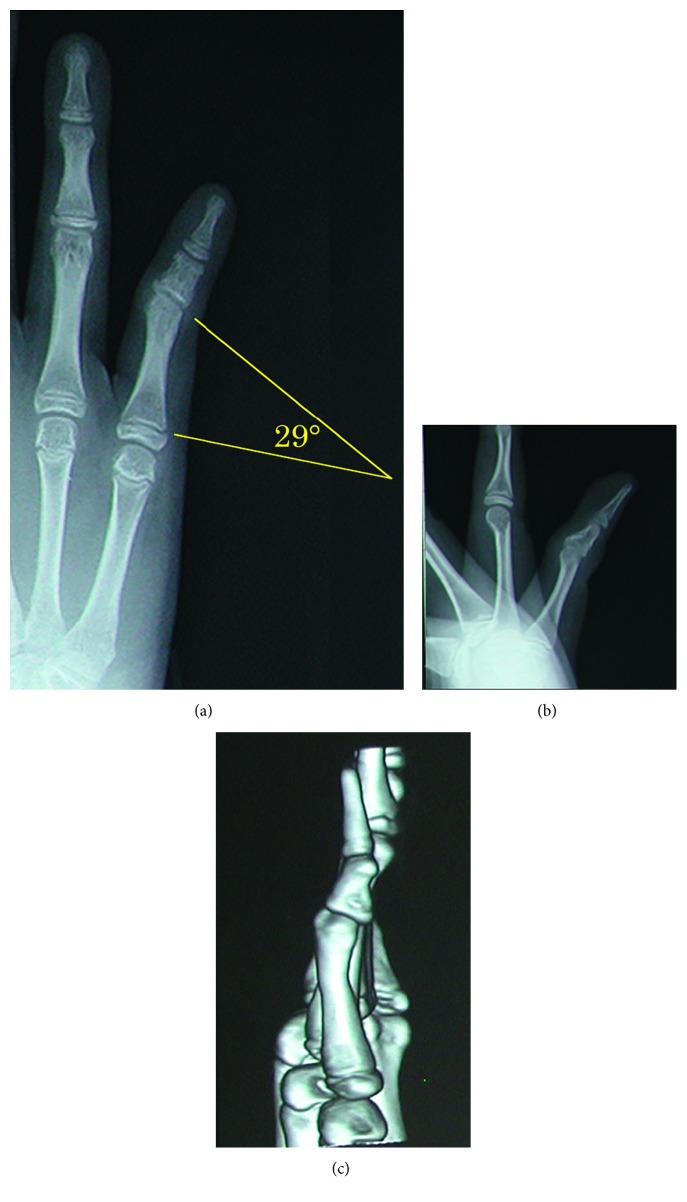
Preoperative imaging in Case 1. (a) Plain radiographic anteroposterior view of the right little finger. The distal articular surface of the proximal phalanx of the little finger was tilted in the ulnar direction. (b) Plain radiographic lateral view of the right little finger. (c) Three-dimensional computed tomography of the proximal phalanx of the right little finger.

Two years after the injury, she consulted our clinic due to functional and cosmetic impairments. There was a gap between the ring and little fingers in the fully extended and adducted position. The patient could not make a fist because of the overlapping of the little finger over the ring finger (Figure 2). The ROM of the finger joints was normal, except for the metacarpophalangeal (MCP) joint of the little finger (right: 40–45°; left: 40–70°). Plain radiography showed that the angle between the proximal growth plate and the distal articular surface of the proximal phalanx was 29° on the affected side compared with 5.5° on the contralateral noninjured side (Figure 3). Bilateral middle brachyphalangia of the little fingers was also detected. Surgery was performed as described above.

**Figure 4:**
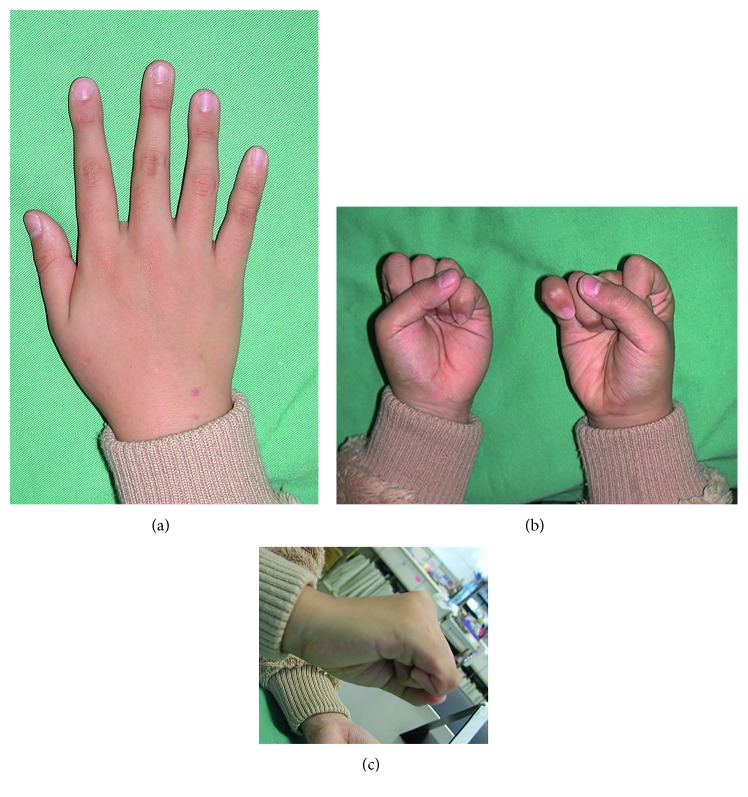
Photographs of Case 1 taken 1 year postoperatively. (a) Dorsal view. (b) Palmar view of the clenched fist. The alignment of the little finger was good. Because of the retained extension contracture of the metacarpophalangeal joint, the tip of the little finger could not touch the palm. (c) Lateral view.

**Figure 5:**
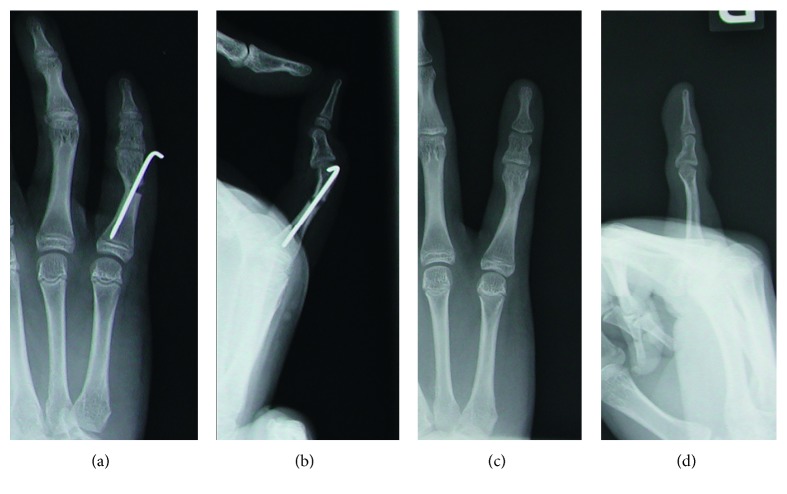
Postoperative radiographs of Case 1. (a, b) Images taken immediately after the surgery. (c, d) Images taken 1 year postoperatively.

At postoperative 1 year, the valgus deformity and overlapping of the little finger had completely resolved (Figure 4). The extension contracture of the little finger MCP joint remained. The angle between the growth plate and the distal articular surface of the proximal phalanx was 4.5° (Figure 5).

### 2.3. Case 2: An 11-Year-Old Female

At 9 years of age, the patient fractured the proximal phalanx of the left little finger in a fall. At the time of the injury, the former doctor performed conservative therapy with cast fixation. Valgus finger deformity was noticed after fixation removal, but this was not addressed as there was no functional impairment.

**Figure 6:**
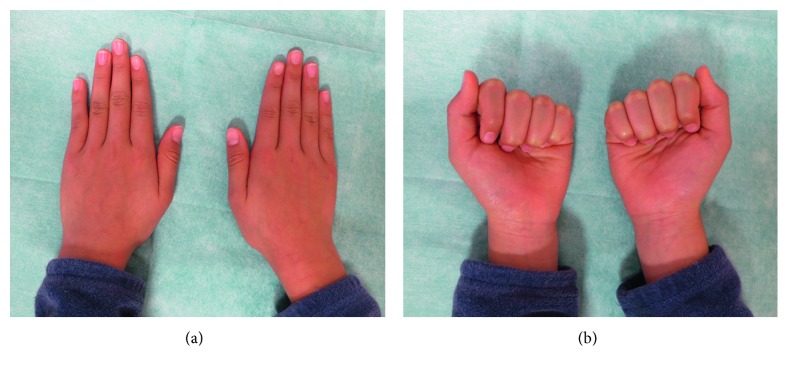
Preoperative photographs of Case 2. (a) There was a gap between the left ring and little fingers in the fully extended and adducted position. (b) The left little finger was partially covered by the ring finger in the fully flexed position.

**Figure 7:**
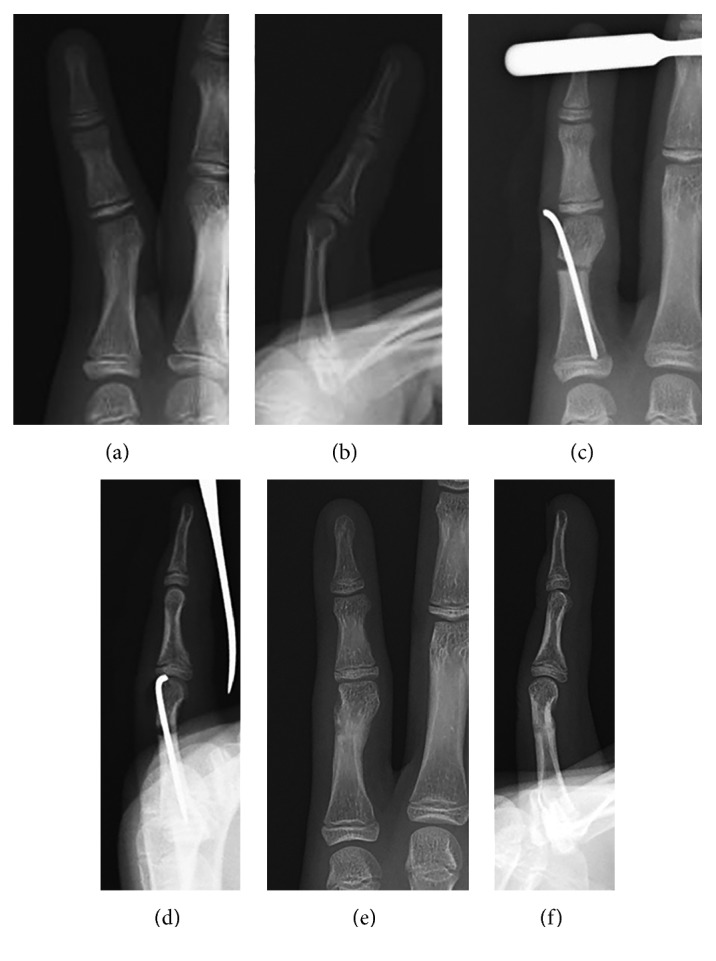
Radiographs of Case 2. (a, b) Preoperative radiography of the left little finger. (c, d) Images taken immediately after the surgery. (e, f) Images taken 3 months postoperatively.

At 2 years after the injury, the patient consulted our clinic due to a gap between the ring and little fingers in the fully extended and adducted position (Figure 6). There was also partial overlapping of the ring finger over the little finger in the fully flexed position. The ROM of the fingers was normal. Plain radiography showed that the angle between the proximal growth plate and the distal articular surface of the proximal phalanx was 15.3° on the affected side compared with 4.7° on the contralateral noninjured side (Figure 7). Surgery was performed as described above.

**Figure 8:**
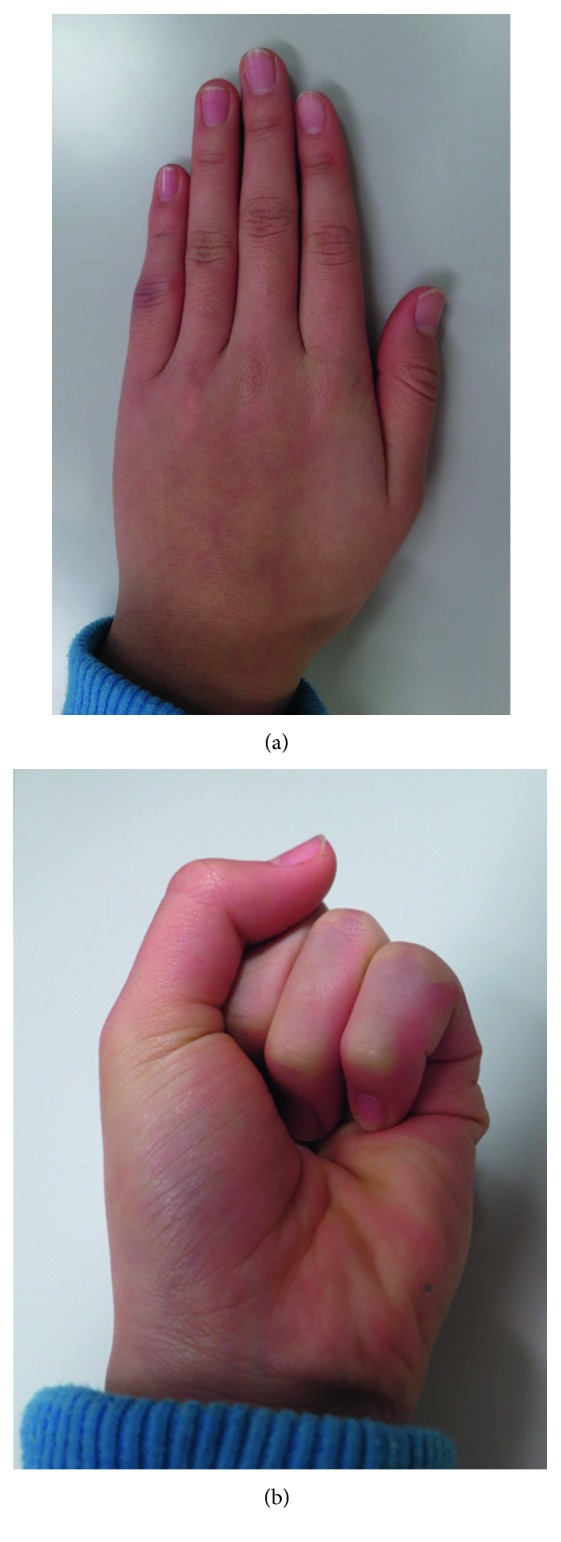
Photographs of Case 2 taken 1 year and 2 months postoperatively. The valgus deformity and overlapping of the left little finger had completely resolved.

At 1 year and 2 months postoperatively, the valgus deformity and overlapping of the little finger had completely resolved (Figure 8). The angle between the growth plate and the distal articular surface of the proximal phalanx was 5.0°.

## 3. Discussion

We described two cases of pediatric little finger valgus deformity successfully treated using opening wedge osteotomy and intramedullary semirigid fixation with a single K-wire.

Closing wedge osteotomy results in a good outcome in correction of an angulated phalanx [3]; however, it is not easy to perform the exact resection of the wedge bone, especially the apex of the wedge bone, according to the preplanned correction angles. Moreover, rigid fixation with a plate or two K-wires makes it impossible to finely adjust the alignment after the fixation procedure. In fixation using two K-wires, the prominent radial stump of the K-wire interferes with the attaching of the little and ring fingers to each other and makes it difficult to check the exact alignment of the little finger. In our procedure, the operative field and the K-wire stump are on the ulnar side of the little finger; hence, there is nothing between the little and ring fingers, and it is easy to check the coronal alignment in the fully extended and adducted position and the rotational alignment in the fully flexed position.

Before the osteotomy, we inserted a K-wire from the retrocondylar fossa of the proximal phalanx at an angle of approximately 20° against the ulnar side cortex of the proximal phalangeal head. This angle is appropriate for further advancement of the K-wire after opening of the osteotomy site; the K-wire tip could be advanced while maintaining contact with the inner cortex without cortical penetration. A K-wire insertion angle of less than 10° should be avoided, as the K-wire may not maintain contact with the radial inner cortex, which will result in undercorrection in the coronal plane. Overcorrection in the coronal plane is prevented by the closely attached normally aligned ring finger and the flexibility of the inserted K-wire.

Correction of the tilted distal articular surface in the coronal plane usually improves the rotational alignment of the little finger to some extent. However, further adjustment of the residual rotational alignment is sometimes needed. In our procedure, further adjustment of the rotational alignment can be performed, even after K-wire insertion; the clearance between the K-wire and the inner wall of the proximal phalangeal medullary cavity in the sagittal plane makes it possible to finely adjust the alignment of the little finger. When fine rotational correction is needed, only gentle manual corrective force should be applied toward the desired direction. As the additional rotation angle required is usually small, further correction is accomplished without disruption of the preserved radial cortex of the osteotomy site. The corrected alignment is maintained during early active exercise with a loosely buddy-taped ring finger.

In the cutting of the bone, multiple drilling was first performed without penetrating the most radial cortex. Bone cutting was then gradually performed with a small osteotome, with pushing performed by hand. Tapping the osteotome with a hammer should be avoided, as fine tuning of the tapping power is difficult.

Opening wedge osteotomy and intramedullary semirigid fixation with a single K-wire allow fine correction of rotational alignment even after K-wire insertion and are appropriate for treating pediatric little finger posttraumatic valgus deformity.
